# Effects of azithromycin on *Pseudomonas aeruginosa* isolates from catheter-associated urinary tract infection

**DOI:** 10.3892/etm.2014.2120

**Published:** 2014-12-08

**Authors:** ZHI-GANG XU, YU GAO, JIAN-GUO HE, WEI-FENG XU, MEI JIANG, HUAN-SHENG JIN

**Affiliations:** 1 Urologic Institute of Chongqing Red Cross Hospital, Chongqing 400020, P.R. China; 2 Urologic Institute of Southwest Hospital, Third Military Medical University, Chongqing 400038, P.R. China

**Keywords:** *Pseudomonas aeruginosa*, azithromycin, urinary tract infection, biofilm, swimming, virulence factor

## Abstract

*Pseudomonas aeruginosa* is a common pathogenic bacterium in urinary tract infections (UTIs), particularly catheter-associated UTIs. The aim of this study was to investigate the effect of azithromycin (AZM) on *P. aeruginosa* isolated from UTIs. Isolates were identified by biochemical assays and the Vitek system. Antimicrobial susceptibility was determined using the disk diffusion assay. Biofilm formation and adhesion were assayed using a crystal violet staining method. The swimming motility was assayed on agar plates. The elastase activity and rhamnolipid production were determined by the elastin-Congo red method and orcinol reaction, respectively. A total of 32 bacterial isolates were collected from 159 urinary catheters and eight of them were *P. aeruginosa* isolates. The results showed that the *P. aeruginosa* isolates had stronger biofilm formation capability and the biofilms were thicker than those of *P. aeruginosa* PAO1. AZM inhibited biofilm formation and adhesion on urinary catheters, and also decreased swimming motility and the production of virulence factors. The results of this study indicated that AZM is potentially a good choice for use in the treatment of UTIs.

## Introduction

Urinary tract infections (UTIs) are among the most common infections in both outpatient and inpatient settings. *Pseudomonas aeruginosa* is a common pathogenic bacteria isolated from UTIs, particularly catheter-associated UTIs (1,2). With the development of medical technology, urinary catheters are applied to greater numbers of people and the time of application is longer. Regarding hospitalized patients, 25% of patients undergo short-term urinary catheterization (<7 days), which increases the risk of developing an infection. Moreover, the UTI rate could reach 100% in hospitalized patients with long-term catheterization (≥30 days) (3). Once the bacterial biofilm develops, the bacterial cells are able to withstand host immune responses, and they are much less susceptible to antibiotics than their nonattached individual planktonic counterparts (4). Due to multiple resistance mechanisms, the higher resistance is more challenging to the clinician. Therefore, numerous researchers have studied the formation, regulation and resistance of biofilms (5–7).

Macrolides have been shown to have a good effect on *P. aeruginosa* infection in the airway tract. However, they are not a good choice for UTI infections (8), and there is little research concerning the effect of macrolides on UTIs. Therefore, in the present study, *P. aeruginosa* isolates were collected from the urinary catheters of hospitalized patients and the *P. aeruginosa* isolates from the urinary catheters was characterized. In addition, azithromycin (AZM) was selected as a representative macrolide to investigate its effect in treating UTIs.

## Materials and methods

### Bacterial strains

Urinary catheters that were applied in hospitalized patients for more than one week were carefully collected under aseptic conditions. The catheters used longer than 7 days were collected and cut open, and a cotton swab was used to scrape the inner wall of possible biofilm infection. The cotton swab was sent immediately to the laboratory for analysis. Isolates were identified by biochemical assays and using the Vitek system (bioMérieux Vitek, Hazelwood, MO, USA). *P. aeruginosa* PAO1 (American Type Culture Collection, Manassas, VA, USA) was used as the control strain and preserved in the laboratory. The *P. aeruginosa* isolates were examined in the following experiments. The present study was conducted according to the principles of the Declaration of Helsinki (2008), and the experimental protocol was approved by the Ethics Committee of Chongqing Red Cross Hospital (Chongqing, China) (approval number, KY201411). Informed consent was obtained from all patients and their families prior to the collection of urinary catheter samples.

### Antimicrobial susceptibility testing

Antimicrobial susceptibility was determined according to the standards of the Clinical and Laboratory Standards Institute (CLSI, 2011) (9) using the Kirby-Bauer disk diffusion assay on freshly prepared *P. aeruginosa* test medium [Müller-Hinton (MH)]. *P. aeruginosa* ATCC27853 and *Escherichia coli* ATCC25922 (American Type Culture Collection) were used as control strains for minimum inhibitory concentration (MIC) testing. The resistance rate was calculated as the number of resistant strains divided by the total number of strains. The susceptible strains included those with susceptibility and medium sensitivity according to the CLSI standards.

### Biofilm assay

The static biofilm assay was performed as outlined by Wang *et al* and Naik *et al* (10,11). In brief, the *P. aeruginosa* strains were grown overnight in Luria-Bertani (LB) broth and diluted to 1×10^6^ CFU/ml with fresh LB. The inoculated culture (150 μl) was transferred to a 96-well polystyrene microtiter plate and incubated at 37°C for 24 h. The planktonic cells were removed from the wells after incubation and the wells were washed three times with sterile water. This was followed by staining of the wells with 0.1% crystal violet for 10 min and washing the unbound stain thrice with sterile water. The cell-bound dye was extracted with 300 μl 95% ethanol, and the absorbance of the solution was measured using a Multiskan Spectrum microplate reader at 595 nm (Thermo Fisher Scientific, Vantaa, Finland).

### Adhesion on urinary catheters

The adhesion on urinary catheters was assayed using the standard crystal violet staining method with a few revisions (12). In brief, a 3-cm urinary catheter was placed into a 6-well board with 4.5 ml LB broth, and 50 μl bacterial culture (1×10^6^ CFU/ml) was added to the well. After incubation for 6 h, unattached cells were removed with sterile water, and attached cells were stained with 1% crystal violet for 20 min. The dye bound to the adherent cells was then solubilized with ethanol-acetone (75:15, v/v) and the optical density of the solution was measured at 570 nm.

### Swimming motility

The swimming motility was assayed using the method of Bala *et al* (13). Bacterial strains were incubated at 37°C overnight. Swimming plates containing 1% tryptone, 0.5% NaCl and 0.3% agar were prepared for the assay. The plates with and without 1/4 MIC AZM were point-inoculated with a sterile toothpick and incubated at 37°C for 24 h. The zone diameter was measured to assess the swimming motility.

### Quantitative analysis of virulence factor production

#### Determination of elastase activity

Elastase activity was determined with elastin-Congo red (ECR) and the steps were as follows: *P. aeruginosa* strains were inoculated onto MH agar plates, and incubated at 37°C for 18 h. A single colony was transferred into 2 ml peptone tryptic soy broth (PTSB) medium, and then the bacterial cultures were transferred into 18 ml PTSB medium with and without 1/4 MIC AZM once the OD_540 nm_ reached 0.5. After incubation for 16 h at 37°C with shaking at 250 rpm, the cultures were centrifuged at 12,100 × g for 15 min at 4°C and the supernatant was filtered with 0.45 μm syringe filter. Then, 1 ml ECR reaction buffer (ECR 20 mg, 0.1 M Tris-HCl/1 mM CaCl_2_, pH 7.2) was added to 1 ml filtered supernatant. When the mixture had been incubated at 37°C for 18 h with shaking at 250 rpm, 0.1 ml 0.12 M EDTA was added to stop the reaction. The reactant was placed on ice and the insoluble ECR was removed by centrifugation at 3,000 × g, 4°C. The elastase activity was determined at 495 nm. Three samples of each type were examined and the experiment was repeated three times.

#### Determination of rhamnolipids

The concentration of rhamnolipid was determined spectrophotometrically by the orcinol reaction using rhamnose as a standard. The orcinol reagent [0.19% orcinol in 53% (v/v) sulfuric acid] was prepared immediately prior to use. The reaction mixture, composed of 100 μl sample (0, 50, 100, 200 and 300 μg/ml) and 900 μl reagent, was well stirred, warmed for 30 min at 80°C, and then kept for 15 min at room temperature. The absorbance was measured at 421 nm. A standard curve was constructed according to the absorbance and concentration of rhamnose.

*P. aeruginosa* strains were grown on LB agar plates at 37°C for 18 h. A single colony was transferred into LB broth and incubated overnight at 37°C with shaking. Then, the OD_600nm_ of the bacterial culture was adjusted to 0.05 with proteose peptone glucose ammonium salts (PPGAS) medium (0.02 M NH_4_Cl, 0.02 M KCl, 0.12 M Tris-HCl, 0.0016 M MgSO_4_, 1% peptone and 0.5% glucose). Bacterial cultures (20 ml) with and without 1/4 MIC AZM were incubated for 48–72 h at 37°C with shaking at 200 rpm. Rhamnolipids were purified by first separating the cells from the supernatant by centrifugation at 6,000 × g for 10 min. The supernatant was then acidified using 12 M hydrochloric acid to pH 2.0, and the precipitated rhamnolipids were collected by centrifugation at 12,100 × g for 5 min. Rhamnolipids were extracted twice with ethyl acetate, which was then evaporated away leaving behind relatively pure rhamnolipids. The rhamnolipids were dissolved in 0.5 ml ddH_2_O and stored at 4°C. For each sample, 100 μl rhamnolipids were determined spectrophotometrically using this method. The content of rhamnose was calculated on the basis of the standard curve. The concentration of rhamnolipid was calculated based on the assumption that 1 μg rhamnose corresponds to 2.5 μg rhamnolipid.

## Results

### Bacterial strains and susceptibility

A total of 159 urinary catheters were collected and 32 showed positive bacterial cultures. Six urinary catheters had more than two kinds of bacteria. Eight *P. aeruginosa* isolates were collected from the urinary catheters. The resistance rates of the eight *P. aeruginosa* isolates to amikacin, ciprofloxacin, levofloxacin, minocycline, ceftazidime, cefotaxime, piperacillin, meropenem, netilmicin, tetracycline and cefepime were 87.5, 87.5, 75.0, 62.5, 87.5, 75.0, 100.0, 62.5, 75.0, 87.5 and 75.0%, respectively. The 1/4 MIC values of AZM on the isolates of *P. aeruginosa* are presented in Table I. The 1/4 MICs ranged from 32 to 256 μg/ml.

### Biofilm formation and adhesion

The effects of AZM on the biofilm formation and adhesion of the *P. aeruginosa* isolates are shown in Table I. The strains from urinary catheters had stronger biofilm formation capability, and the biofilms of eight isolates were thicker than those of *P. aeruginosa* PAO1 (data not shown) The 1/4 MIC AZM may reduce the biofilm formation capability and the adhesion to urinary catheters.

### Swimming motility

**Figure 1:**
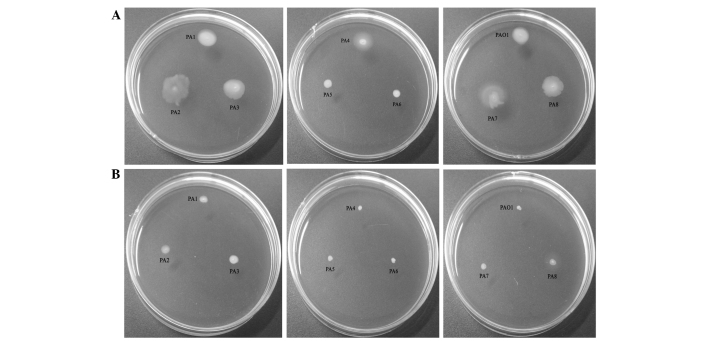
Demonstration of the swimming motility of standard strain PAO1 and *P. aeruginosa* clinical isolates PA1-PA8 (A) in the absence (control group) and (B) presence (tested group) of azithromycin.

The results of the assay investigating the effects of AZM on swimming motility are presented in Fig. 1. It was observed that the presence of AZM significantly reduced the motility of all *P. aeruginosa* strains.

### Virulence factors

**Figure 2:**
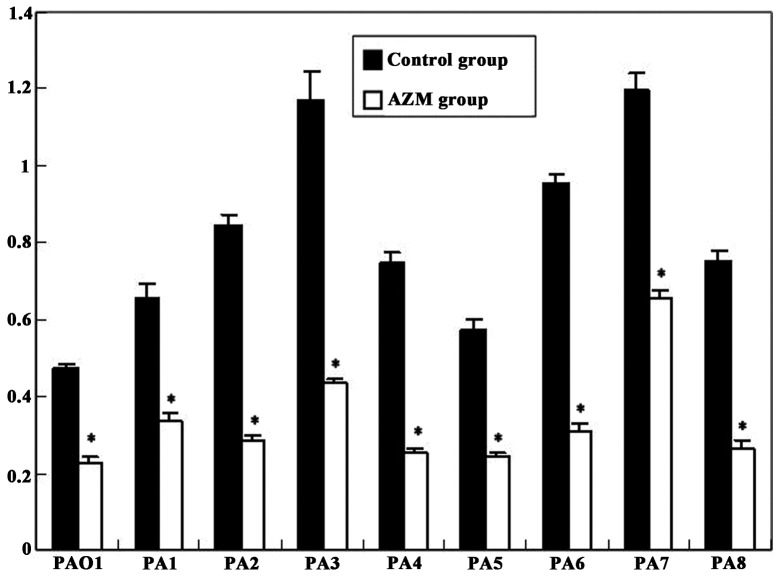
Elastase activity of *P. aeruginosa* standard strain PAO1 and isolates PA1-PA8. ^*^P<0.01, compared with the control group.

**Figure 3:**
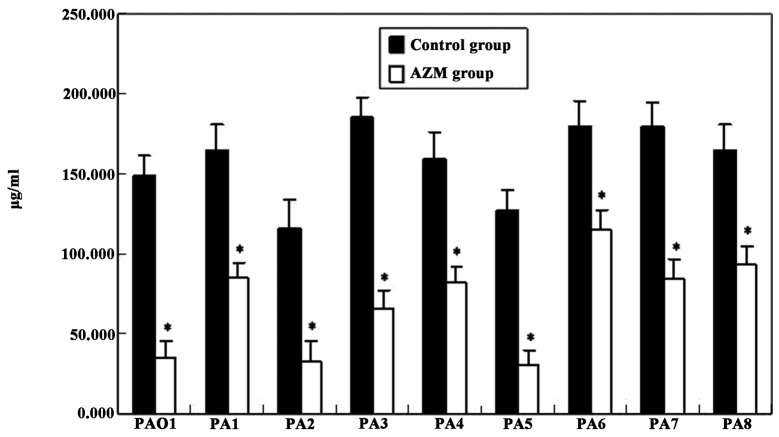
Rhamnolipid production by of *P. aeruginosa* standard strain PAO1 and isolates PA1-PA8. ^*^P<0.01, compared with the control group.

The elastase activity of all *P. aeruginosa* clinical isolates was higher than that of PAO1 in the control and AZM groups, respectively (Fig. 2). The production of rhamnolipids by the *P. aeruginosa* clinical isolates was higher than that by PAO1, with the exception of isolates PA2 and PA5 (Fig. 3). Moreover, the presence of AZM in the culture medium greatly decreased the expression of these virulence factors (P<0.01; Figs. 2 and 3).

## Discussion

With the development of medical technology, urinary catheterization is increasingly common in hospitalized patients, which is a risk factor of bacteriuria and symptomatic UTI (14). In the present study, the rate of isolation of positive strains was 20%, and the *P. aeruginosa*-positive rate was 5%. Among the eight obtained isolates, the biofilms were thicker than those of *P. aeruginosa* PAO1, which suggested that the strains isolated from the urinary catheters had stronger biofilm formation capability. Therefore, for *P. aeruginosa* in UTI, the possibility of biofilm formation during therapy should be considered. Swimming motility is a significant etiological factor. In the presence of 1/4 MIC AZM, the swimming motility was depressed greatly. Moreover, AZM not only decreased biofilm formation capability, but also decreased the adhesion to urinary catheters, which is similar to the findings of previous studies (13,15).

*P. aeruginosa* elastase (PE) is a 39.5-kDa metalloproteinase and one of the strongest virulence factors of *P. aeruginosa*, which can degrade the elastin of human matrix proteins including laminin and collagen types III and IV (16–18). PE has also been found to be an immunosuppressive factor (19,20). In biofilm formation, rhamnolipids have important actions, which are involved in modulating the swarming and colonization of incipient biofilm forming (21). The inhibitory effect of AZM on the production of rhamnolipids was less significant than its inhibitory effect on elastin. However, the rhamnolipid levels were markedly decreased. The results demonstrated that AZM decreased the elastase activity greatly, indicating that AZM might relieve the pathogenicity of *P. aeruginosa* from UTIs.

AZM has been shown to exert a good therapeutic effect on cystic fibrosis (22). However, to the best of our knowledge, there are few reports concerning the use of AZM in the treatment of UTIs. Administration of prophylactic antibiotics following catheter application decreases UTI rates (23). However, prophylactic antibiotics are usually trimethoprim-sulfamethoxazole and quinolones (23). Macrolides are not a good choice for UTIs according to the clinical application principles of antibiotics of China (8). As AZM is a new macrolide antibiotic, it is inferred from the present study results that macrolides may be a good choice in the treatment of UTIs involving *P. aeruginosa*. However, there are many factors affecting the clinical treatment effect, such as the immunity condition of patients, the original disease and resistance of *P. aeruginosa*. Therefore, it is necessary to conduct further clinical studies.

## Figures and Tables

**Table I:** Effect of 1/4 MIC azithromycin on the biofilm formation and adhesion of *P. aeruginosa*.

Variable	PAO1	PA1	PA2	PA3	PA4	PA5	PA6	PA7	PA8
1/4MIC	64	128	64	64	128	64	256	32	64
OD_biofilm_	0.46	0.33	0.48	0.32	0.66	0.27	0.68	0.37	0.39
OD_adhesion_	0.43	0.52	0.47	0.44	0.36	0.36	0.31	0.61	0.47

MIC, minimum inhibitory concentration; OD, optical density. OD_biofilm_ and OD_adhesion_ indicate the ratios of OD values between the 1/4 MIC AZM group and the control group. PAO1, control strain; PA1–8, *P. aeruginosa* clinical isolates.
